# Pig as a reservoir of CRISPR type TST4 *Salmonella enterica* serovar Typhimurium monophasic variant during 2009–2017 in China

**DOI:** 10.1080/22221751.2019.1699450

**Published:** 2019-12-20

**Authors:** Xiaolei Xie, Zhenyu Wang, Kai Zhang, Yang Li, Yachen Hu, Zhiming Pan, Xiang Chen, Qiuchun Li, Xinan Jiao

**Affiliations:** a Key Laboratory of Prevention and Control of Biological Hazard Factors (Animal Origin) for Agri-food Safety and Quality, Ministry of Agriculture of China, Yangzhou University, Yangzhou, People’s Republic of China; b Jiangsu Key Laboratory of Zoonosis/Jiangsu Co-Innovation Center for Prevention and Control of Important Animal Infectious Diseases and Zoonoses, Yangzhou University, Yangzhou, People’s Republic of China; c Joint International Research Laboratory of Agriculture and Agri-Product Safety of Ministry of Education of China, Yangzhou University, Yangzhou, People’s Republic of China

**Keywords:** *Salmonella enterica* serovar Typhimurium (*Salmonella* Typhimurium), *Salmonella* 4,[5],12:i:-, CRISPR typing, pig, human

## Abstract

CRISPR-based typing was performed to subtype isolates of *S.* Typhimurium and its monophasic variant *Salmonella* 4,[5],12:i:- from humans and animals between 2009 and 2017 in China. CRISPR typing classified all isolates into two lineages and four sub-lineages. All isolates from Lineage II and Lineage IB-1 were *Salmonella* Typhimurium. All of *Salmonella* 4,[5],12:i: – isolates were distributed in Lineage IA and Lineage IB-2, which all belonged to ST34 by MLST typing. Only Lineage IB-2 contained ST34 isolates from both *Salmonella* Typhimurium and *Salmonella* 4,[5],12:i:-. Among the isolates of ST34, TST4 was identified as the most common CRISPR type representing 86.5% of *Salmonella* 4,[5],12:i:- and 14.5 % of *Salmonella* Typhimurium mainly from pigs and humans. This study demonstrated that TST4-ST34 isolates were predominant in *Salmonella* 4,[5],12:i:-, and pig was the main reservoir for *Salmonella* 4,[5],12:i:- in China, which might have the potential to transmit to humans by pig production.

*Salmonella enterica* serovar Typhimurium (*Salmonella* Typhimurium) is one of the most important zoonotic pathogens causing food-borne gastroenteritis across the world [1,2] Human infections with *Salmonella* Typhimurium are typically associated with contaminated food of animal origin [3]. Recently, a *Salmonella* Typhimurium monophasic variant (*Salmonella* 4,[5],12:i:-) has been increasingly isolated from husbandry animals, foods, and humans [4]. Among the common serovars associated with human salmonellosis cases in Europe, the monophasic *Salmonella* Typhimurium has ranked third after *Salmonella* Enteritidis and *Salmonella* Typhimurium in 2017 [5]. In USA, the *Salmonella* 4,[5],12:i:- was confirmed to be the most increased serotype from 1972 to 2016, and stayed as the top 5 serotype for human salmonellosis during 2011 and 2016 [6]. However, few reports described the prevalence of *Salmonella* 4,[5],12:i:- in human salmonellosis in China. In 2015, 13 foodborne isolates of *Salmonella* 4,[5],12:i:- were firstly reported in Guangdong province. A recent study pointed out that *Salmonella* 4,[5],12:i:- had increased to be the second most frequently encountered serotype in patients in Henan province, China [7]. Comparative analysis of genome sequences and biological properties has revealed that deletion or mutation of the *fljB* gene causes loss of phase 2 flagellin expression in the monophasic variant [8]. And the MLST type was not an efficient tool to differentiate them [9]. Therefore, new efforts are needed to demonstrate the genetic and phenotypic difference between *Salmonella* Typhimurium and its monophasic variant with prevalence characteristics of both serotypes. It is also important to understand the phylogenetic relationship between the two serotypes in order to develop new eradication strategies.

The Clustered Regularly Interspaced Short Palindromic Repeats (CRISPR) typing has been used as a high-resolution typing method of a broad range of bacteria. Thus far, CRISPR typing has been widely used to subtype *Salmonella* isolates belonging to identical serotypes including *Salmonella* Typhimurium, *Salmonella* Enteritidis, and *Salmonella* Pullorum [10–14]. Such studies have demonstrated that CRISPR typing is efficient in discriminating isolates from different sources and time periods. Further, the arrangement and microevolution of CRISPR spacers allows typing and subtyping to be performed in a single step. In the present study, we used CRISPR typing to identify genotypic relationships among 173 isolates of *Salmonella* Typhimurium and *Salmonella* 4,[5],12:i:- obtained from different hosts during 2009–2017 in China. Our findings demonstrate the presence of a predominant CRISPR type shared by these two serotypes in both humans and pigs, and reveal the pig as a main reservoir for *Salmonella* 4,[5],12:i:-, which can also infect human.

We used CRISPR typing to genotype 173 isolates of *Salmonella* Typhimurium (62) and its monophasic variant *Salmonella* 4,[5],12:i:- (111) obtained from different sources during 2009–2017 in China (Supplementary Table S1). Animal-origin *Salmonella* Typhimurium and *Salmonella* 4,[5],12:i:- isolates were collected from commercial farms, slaughterhouse, and retail markets, while human isolates were collected from diarrhea patients in hospitals. Identification of *Salmonella* 4,[5],12:i:- was performed by slide agglutination with somatic (O) and flagellar (H) antiserum combined with multiplex-PCR approach of *fliB-fliA* intergenic region and the *fljB* gene, respectively [8]. CRISPR typing was performed as previously described [15]. Among the 173 isolates, 67 unique spacers were detected in the two CRISPR loci, with 31 in CRISPR1 and 36 in CRISPR2. Based on the spacer arrangement, 30 different alleles were observed in CRISPR1, and 16 different alleles in CRISPR2 (Figure 1(A)). With the combination of CRISPR1 and CRISPR2 arrays, a total of 34 different Typhimurium CRISPR types were identified and named using a number suffix to TST as previously indicated (Figure 1(A,B)). Cluster analysis using UPGMA revealed that only seven out of the 34 TSTs shared between isolates from different host. TST4, a combination of CRISPR1 allele 7 and CRISPR2 allele 6, was found to be the most frequent CRISPR type shared by 55% (96/173) of the isolates (Figure 1(B)). TST4 and TST17 were common among isolates from pigs, humans and chicken, which showed the potential transmission between animals and humans. TST20, TST27, TST30, TST31, and TST33 were only detected in isolates of poultry origin and were distant from TSTs of other origins. Two isolates collected from cattle belonged to TST9. This revealed that CRISPR types also reflected the source of isolates [16]. TST4 isolates were observed from six out of nine provinces demonstrating its predominant prevalence in China (Supplementary Table S1). The second most common CRISPR type TST5 was detected in three provinces with only 11 isolates. In Jiangsu province, 13 CRISPR types were detected in 38 isolates from Yangzhou city, but only 4 CRISPR types in 27 isolates from Huaian city. These findings reflected that CRISPR types were closely related to different regions.

**Figure 1.**
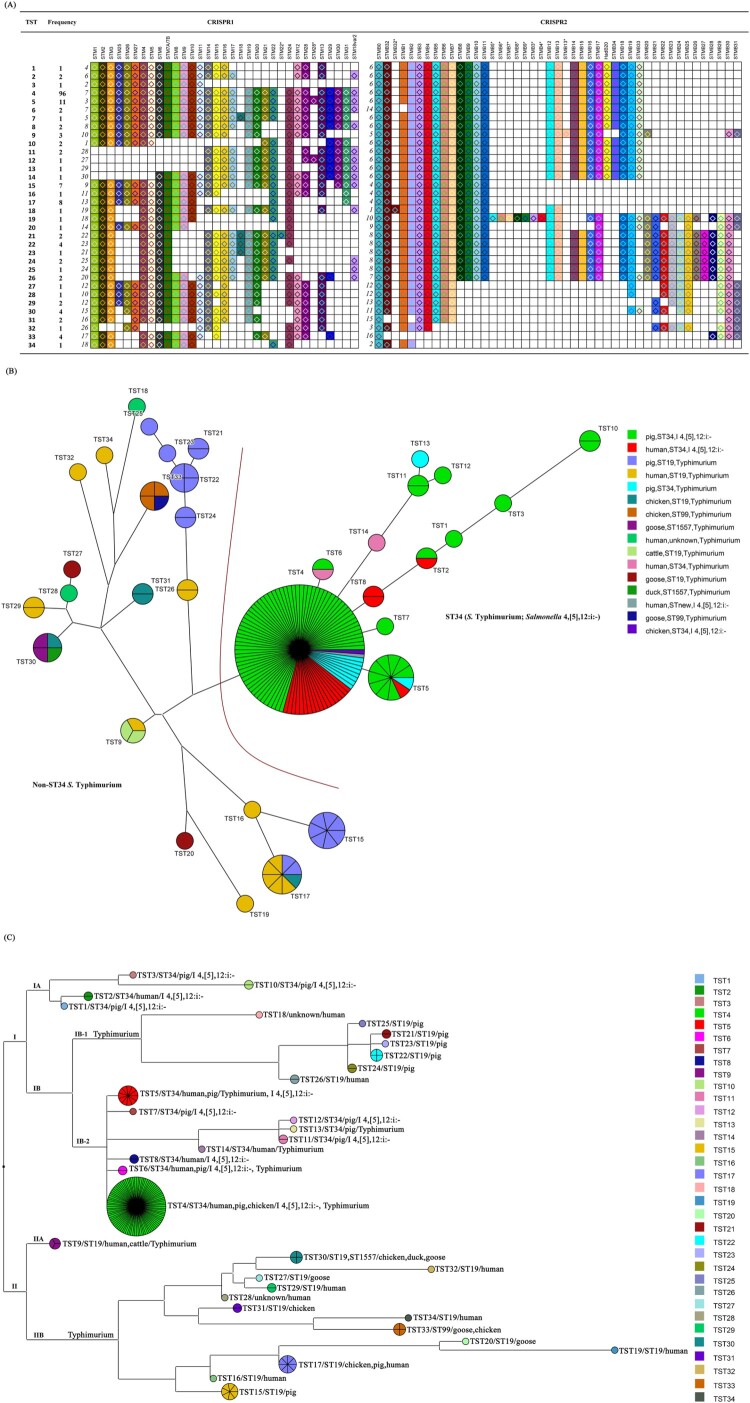
The CRISPR type and phylogenic relationship of 173 *Salmonella* Typhimurium and its monophasic variant isolates. (A) The CRISPR type for each strain is represented by spacers arrangement in CRISPR1 (left) and CRISPR2 (right) locus. Each unique spacer is represented by a unique combination of background colour with the colour and shape of the object in the foreground. Name of the spacers are labelled on the top of each spacers. And the frequency of each allelic type is listed on the left side of the CRISPR allele. The TSTs (Typhimurium sequence types) are derived from the combination of CRISPR 1 and CRISPR alleles. (B) The minimum spanning phylogenetic tree built from the CRISPR allelic profiles of 173 *Salmonella* Typhimurium and *Salmonella* 4,[5],12:i:- isolates. The size of each circle is proportional to the number of isolates in this circle and isolates in the same circle share the same CRISPR type. All TSTs are labelled and the origins of isolates are indicated with unique colours on the right side. The left side is composed of non-ST34 *Salmonella* Typhimrium isolates, while the right side includes only ST34 isolates of *Salmonella* Typhimurium and *Salmonella* 4,[5],12:i:- isolated mainly from humans and pigs. (C) The maximum-parsimony approach was performed to reveal the genetic relationship of 34 TSTs. Two lineages and four sub-lineages clearly showed that Lineage II and IB-1 was only *Salmonella* Typhimurium isolates, while Lineage IA was occupied by *Salmonella* 4,[5],12:i:-. Only Lineage IB-2 was mixed by both serotypes.

Compared with MLST type of the 173 isolates, the CRISPR typing divided the 122 ST34 isolates into 14 TSTs (Figure 1(A,B)), which confirmed that CRISPR typing has stronger discriminatory power than MLST [17]. As shown in Figure 1(B), 95 out of 96 TST4 strains shared the ST34 type, including isolates of both *Salmonella* Typhimurium and *Salmonella* 4,[5],12:i:-, mostly from pigs and humans. However, all of the eight (12.9%, 8/62) TST4-ST34 *Salmonella* Typhimurium strains were isolated from pigs, while the 88 (79.3%, 88/111) TST4-ST34 *Salmonella* 4,[5],12:i:- strains included 68 pig isolates, 19 human isolates, and one chicken isolate, suggesting that *Salmonella* 4,[5],12:i:- has become more frequently transmitted to human through contaminated food than *Salmonella* Typhimurium. Whole genome sequencing analysis of *Salmonella* Typhimurium and its monophasic variant from Denmark demonstrated that ST34 was the main MLST type in the monophasic variant isolates, which was also shared by *Salmonella* Typhimurium isolated from humans, food, and veterinary samples [9]. In the present study, we not only confirmed that ST34 is predominant among *Salmonella* 4,[5],12:i:- isolates, but also demonstrated that TST4 is the main CRISPR type shared by both serotypes among these ST34 isolates, which were mainly from swine or pork meat (Figure 1(B)). Apart from TST4-ST34, which was shared by both serotypes, TST5-ST34 and TST6-ST34 were also shared by the two serotypes isolated from both pigs and humans (Figure 1(B)).

According to data reported by the European Food Safety Authority (EFSA) and the European Centre for Disease Prevention and Control (ECDC), *Salmonella* Typhimurium ranked second after *Salmonella* Enteritidis and followed by its monophasic variant serovar associated with human salmonellosis cases in Europe during 2017 [5]. In addition, 39.4% of *Salmonella* Typhimurium isolates and 81.4% of its monophasic variant isolates from human cases showed multi-drug resistance (MDR), a much higher prevalence of resistance than 28.6% of all *Salmonella* isolates from human salmonellosis [5]. Thus, *Salmonella* Typhimurium and its monophasic variant are considered as a serious epidemic threat to public health with apparent worldwide distribution. Thus far, although swine or pork has been considered as the main source of infection in many countries, the genetic relationship between *Salmonella* Typhimurium and *Salmonella* 4,[5],12:i:- is not well understood. To identify the genetic relationship between the two serotypes, a phylogenic tree was constructed based on the 34 identified TSTs (Figure 1(C)) using Bionumericus 7.5. As shown in Figure 1(C), they are divided into two main lineages and four sub-lineages. Interestingly, Lineage IA was found to be composed of TSTs specific to *Salmonella* 4,[5],12:i:- isolates, while Lineage II and IB-1 was exclusively composed of TSTs specific to *Salmonella* Typhimurium strains mainly of the ST19 type. Interestingly, only Lineage IB-2 contained TSTs shared by both *Salmonella* Typhimurium and *Salmonella* 4,[5],12:i:-. Although there was a low number of isolates with TSTs specific to *Salmonella* Typhimurium or *Salmonella* 4,[5],12:i:-, this diversity reflected evolutionary divergence among the two serotypes. Both *Salmonella* Typhimurium and *Salmonella* 4,[5],12:i:- were observed in TST4, TST5 and TST6, which confirmed a close genetic relationship between these two serotypes.

In addition, CRISPR typing showed higher discriminatory power than PFGE and MLVA, and it could correctly identify all major lineages defined by whole genome single nucleotide polymorphism typing (WGST) of *Salmonella* Enteritidis isolates [18]. However, CRISPR typing could not efficiently delineate outbreak clusters, which could be resolved by WGST in further study.

In conclusion, CRISPR typing has been widely used as a high-resolution typing method based on the fact that genetic diversity of CRISPR sequences can provide valuable insights into microevolution and evolutionary trajectories of bacterial isolates including *Salmonella*. In the present study, we demonstrated that TST4-ST34 strains were predominant in most *Salmonella* 4,[5],12:i:- isolates and shared by some *Salmonella* Typhimurium isolates, obtained from humans, pigs, and chicken. Furthermore, the pig was found to be the main reservoir for these TST4-ST34 isolates, suggesting that the monophasic variant might be produced via mutation of *Salmonella* Typhimurium in pigs. The prevalence of the TST4-ST34 *Salmonella* 4,[5],12:i:- strains in animals should be considered a matter of public health concern, and monitored by the government to prevent transmission to humans.

## Supplementary Material

Supplemental Material
